# Obesity and Depression in Adults: What’s Behind These Growing Epidemics?

**DOI:** 10.1192/j.eurpsy.2026.11438

**Published:** 2026-07-31

**Authors:** S. Bradai, N. Ketata, N. kallel, R. Khmakhem, R. gargouri, S. msaed, H. Ben ayed, N. moussa, N. kallel, J. Jedidi

**Affiliations:** Hedi Chaker hospital, sfax, Tunisia

## Abstract

**Introduction:**

Obesity and depression are major public health concerns due to their high prevalence and related morbidity and mortality.

**Objectives:**

This study aimed to assess the prevalence of obesity, identify associated risk factors, and explore its association with depression among Tunisian adults

**Methods:**

A population-based cross-sectional study was conducted in 2023 among Tunisian-adults aged ≥18 years. Obesity was defined as BMI ≥30 kg/m^2^ and classified into class I (30.0–34.9), class II (35.0–39.9), and class III (≥40.0). Depression was assessed using the the validated Patient-Health-Questionnaire-9(PHQ-9), with scores ≥20 indicating severe depression.

**Results:**

The total of 3,074 participants included 1,941 females (63.1%), 1,504 married individuals (48.9%), 2,716 urban residents (88.4%), and 2,357 with university-level education (76.7%). Chronic diseases were reported in 797 participants (25.9%). The mean age was 36.3 ± 13.6 years. Lifestyle behaviors included energy drink consumption (n=1,554; 50.6%), frequent copious dinners (n=1,335; 43.4%), chronic alcohol use (n=317; 10.3%), active smoking (n=439; 14.3%), and regular physical activity (n=735; 23.9%).

Obesity was found in 378 individuals (12.3%), with 318 in class I (84.1%) and 16 in class III (4.2%). Depression affected 173 participants (5.6%). Obesity was significantly associated with being married (OR=2.4; p<0.001), energy drink consumption (OR=1.3; p=0.02), chronic disease (OR=2.2; p<0.001), copious dinners (OR=1.6; p=0.001), and depression (OR=1.7; p=0.005). University education (OR=0.6; p<0.001) and regular physical activity (OR=0.3; p<0.001) were identified as protective factors against obesity

**Conclusions:**

Obesity was significantly associated to depression as well as unhealthy lifestyle habits. Targeted health interventions are needed to promote healthier lifestyles.

**Disclosure of Interest:**

None Declared

